# Intracranial Mönckeberg’s Atherosclerosis Is Frequently Found in Autopsy Cases of Advanced Stage Malignancy with Cerebral Infarction

**DOI:** 10.3390/cancers13205234

**Published:** 2021-10-19

**Authors:** Chika Shichijo, Keita Kai, Kazuki Jinnouchi, Masashi Nishihara, Hideo Hara, Shinichi Aishima

**Affiliations:** 1 Department of Pathology & Microbiology, Saga University Faculty of Medicine, Saga 849-8501, Japan; 17624007@edu.cc.saga-u.ac.jp (C.S.); sw8696@cc.saga-u.ac.jp (K.J.); saish@cc.saga-u.ac.jp (S.A.); 2 Division of Neurology, Department of Internal Medicine, Saga University Faculty of Medicine, Saga 849-8501, Japan; hihara@cc.saga-u.ac.jp; 3 Department of Pathology, Saga University Hospital, Saga 849-8501, Japan; 4 Department of Radiology, Saga University Faculty of Medicine, Saga 849-8501, Japan; nishiham@cc.saga-u.ac.jp

**Keywords:** cerebral infarction, malignant tumor, Mönckeberg’s atherosclerosis, medial arterial calcification, chronic renal disease

## Abstract

**Simple Summary:**

We pathologically compared the cerebral infarction (CI) status in autopsy cases with and without malignancy focusing on the status of intracranial Mönckeberg’s atherosclerosis. Most of Mönckeberg’s atherosclerosis were found in the basal ganglia. Its prevalence in CI cases with malignancy was significantly higher than in malignancy cases without CI and was apparently higher than CI cases without malignancy. The results indicated that Mönckeberg’s atherosclerosis was frequently found in the basal ganglia of CI patients with malignancy and that intracranial Mönckeberg’s atherosclerosis is a potential risk factor for CI in patients with advanced stage malignancy.

**Abstract:**

Cerebral infarction (CI) severely affects the prognosis of patients with malignancy. The aim of the study was to compare the pathology of CI between cases with and without malignancy focusing on intracranial Mönckeberg’s atherosclerosis. Among 778 autopsy cases of craniotomy, 53 cases of “cerebral infarction without malignancy group” (CI group), 50 cases of “malignant tumor without CI group” (MT group), and 39 cases of “cerebral infarction with malignancy group” (CM group) were identified. Mönckeberg’s atherosclerosis was mainly found in the basal ganglia and its prevalence in the CM group (38.5%) was significantly higher than in the MT group (12.0%, *p* = 0.005), and apparently higher than in the CI group (18.9%, *p* = 0.057). The CI group was significantly older, had higher BMIs, and a greater prevalence of hypertension and atrial fibrillation compared to the CM group. In addition, the prevalence of chronic renal disease was significantly lower in the CM group (2.6%, *p* = 0.012) than in the CI group (20.8%). Our results indicated that Mönckeberg’s atherosclerosis was often found in the basal ganglia of CM cases and that intracranial Mönckeberg’s atherosclerosis is a potential risk factor for CI in patients with advanced stage malignancy.

## 1. Introduction

The incidence of cerebrovascular disorder in patients with malignant tumors is considered to be not so different to that in patients without malignant tumors [1,2,3]. However, stroke in patients with cancer is associated with subsequent unfavorable clinical outcomes [2,4]. It has long been known that patients with malignant tumors often have accompanying coagulation disorders, complicated by thrombus or multiple organ infarctions. Armand Trousseau first suggested a correlation between malignant tumors and migratory thrombophlebitis in 1865 [5] and Sack et al. reported the following disorders in patients with malignancy as “Trousseau syndrome”, namely a combination of microvascular thrombosis, endocarditis, and arterial thrombosis caused by disseminated intravascular coagulation [6]. Subsequently, cancer-related thrombosis has been widely studied, although it should be noted that the definition of “Trousseau syndrome” is ambiguous and remains controversial [7].

Cerebral infarction (CI) severely affects the activities of daily living and the prognosis of patients with malignancy. Lung, gastric, colon, and gynecological cancers have been demonstrated to be the cancers most frequently associated with stroke [8,9], with the most common histological type being adenocarcinoma associated with the production of mucin-enhancing thrombus formation [10]. However, many specific conditions of malignancy might cause CI, such as tumor embolism, coagulation disorders, infections, therapy-related events, and paraneoplastic syndrome [1,10]. The general risk factors for CI include hypertension, regular physical activity, smoking, diabetes, psychosocial, the apolipoprotein (Apo)B/ApoA1 ratio, and cardiac-related causes [11].

Thus, the risk factors for CI differ when a patient has a malignancy. A number of clinical studies compared CI associated with malignancy and CI without malignancy [1,4]. To the best of our knowledge, however, there have been no previous studies that compared the pathology of CI between autopsy cases with and without malignancy.

The initial aim of the present study was to compare the pathology of CI between cases with and without malignancy. We were also interested in intracranial Mönckeberg’s atherosclerosis and also focused on this pathology.

## 2. Materials and Methods

### 2.1. Patients

**Figure 1:**
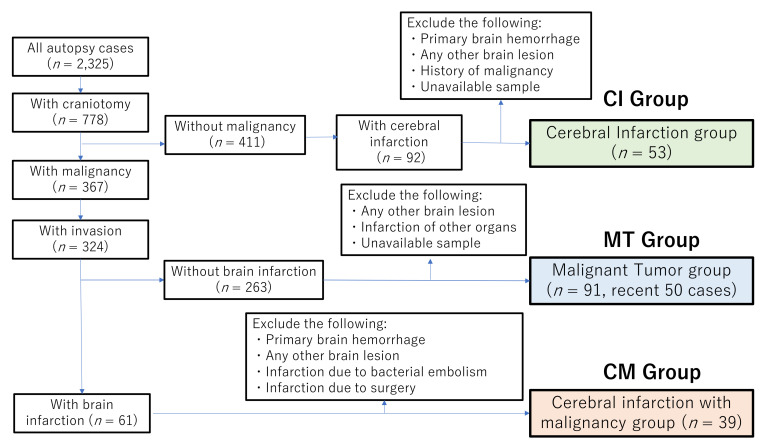
Flowchart for case selection.

A total of 2325 autopsy cases performed between 1981 and March 2019 at Saga University Hospital were initially studied. Of these, 778 cases underwent craniotomy. The flowchart for case selection is shown in Figure 1.

The cerebral infarction group (CI group) was defined as cases in which CI was confirmed by pathological examination of the brain, with the following cases excluded: CI due to tumor embolism; brain tumor; brain metastasis; primary cerebral hemorrhage; meningitis; encephalitis; CI due to postoperative complications; hypoxic ischemic encephalopathy; neurodegenerative disease; and a previous history of malignant tumor. As a result, 54 cases corresponded to these criteria. One case was excluded because the brain tissue was not available and 53 cases were finally included in the CI group.

The malignant tumor group (MT group) was defined as cases with malignant tumors at advanced stages (invaded or metastasized into other organs at autopsy). Excluded cases were: individuals with non-invasive, early stage (without invasion or metastasis into other organs) and latent cancers; CI detected by pathological examination; a history of CI; infarction of other organs; intracranial lesions; and brain surgery cases. As a result, 91 cases were allocated to the MT group. Of these, the 50 most recent cases were included in the present study.

The CI with malignancy group (CM group) was defined as cases with both malignant tumors at an advanced stage and CI confirmed by pathological examination at autopsy. The following cases were excluded: CI due to tumor embolism; brain tumor; brain metastasis; primary cerebral hemorrhage; meningitis; encephalitis; cerebral infarction due to postoperative complications; hypoxic ischemic encephalopathy; and neurodegenerative disease. As a result, a total of 39 cases comprised the CM group.

Written informed consent for usage for research purposes of the clinical data and tissue specimens was obtained from the bereaved families of all patients prior to autopsy. The study protocols were approved by the Ethics Committee of Saga University Hospital (approval number: 2021-03-R-07).

### 2.2. Clinical Data and Pathological Assessments

All clinical information for each case including age, gender, body mass index (BMI) and the clinical history were obtained from medical records. As risk factors for CI, data on the presence or absence of hypertension, diabetes, atrial fibrillation, and chronic kidney disease (CKD) were also collated.

The histology of malignant tumors and CI in autopsy specimens was confirmed using a light microscope by at least two experienced pathologists. Localized necrosis, spongiosis, gliosis, and macrophage infiltration of brain tissue were defined as “histological CI” in the present study and infarct lesions < 1.5 cm were defined as “lacuna infarction”.

Although the number of brain sections varied by case, the following factors of the CI lesions were pathologically thoroughly evaluated: number of CI lesions (single or multiple); hemorrhage (hemorrhagic infarction); and location of CI (cerebral cortex/subcortex, basal ganglia/thalamus; cerebellum; and brain stem).

We also focused on blood vessels (especially arteries) near to the CI. The vessels were examined after hematoxylin and eosin (HE), Elastica van Gieson (EVG), and Congo-red staining. Amyloid deposition was assessed as positive after positive Congo-red staining on light microscopy examination and confirmed by apple-green polarization.

### 2.3. Statistical Analysis

JMP Pro-version 15 software (SAS Institute, Cary, NC, US) was used for all statistical analyses. Normally distributed data are presented as the mean ± standard deviation (SD) and compared between the two groups using a χ^2^ or Fisher’s exact test when appropriate (two-tailed). Count data were compared between groups using Fisher’s exact test (two-tailed). *p*-values < 0.05 were considered to be significant.

## 3. Results

### 3.1. Clinicopathological Characteristics of the CM and CI Groups

The clinicopathological characteristics of the CM and CI groups are summarized in Table 1. The average age of the CM group (69.4 ± 9.5 years) was significantly lower (*p* = 0.009) than that of the CI group (75.0 ± 10.4 years). The CM group was comprised of 22 males (56.4%) and 17 females (43.6%), and the CI group 31 males (58.5%) and 22 females (41.5%). BMIs in the CM group (18.9 ± 0.6) were significantly lower (*p* = 0.019) than in the CI group (21.0 ± 0.6), with no significant difference found between genders.

Regarding the prevalence of clinical risk factors for infarction, such as hypertension, atrial fibrillation, and CKD, they were significantly higher in the CI group (*p* = 0.027, 0.037, and 0.011, respectively), but no significant difference was found regarding the prevalence of diabetes.

Infarction of other organs was detected in 41.0% of the CM group and 58.5% of the CI group with no significant difference between the groups. Infarction of the spleen in the CM group (15.4%) was significantly higher (*p* = 0.039) than in the CI group (1.9%).

### 3.2. Clinicopathological Characteristics of the CM and MT Groups

The clinicopathological characteristics of the CM and MT groups are summarized in Table 2. The mean age of the CM group (69.4 ± 9.5 years) was apparently higher than in the MT group (64.0 ± 14.7 years) although statistical significance was not reached (*p* = 0.052). The MT group comprised 28 males (56.0%) and 22 females (44.0%), with virtually the same population characteristics compared to the CM group. No significant difference was found between the CM and MT groups with regard to BMI, hypertension, diabetes, atrial fibrillation, or CKD.

The most common primary sites in the CM group were the digestive system (*n* = 11, 28.2%), followed by hematopoietic and lymphoid tissues (*n* = 9, 23.1%), lung (*n* = 5, 12.8%), head and neck (*n* = 5, 12.8%), female genital organs (*n* = 4, 10.3%), kidney (*n* = 2, 5.1%), peritoneum (*n* = 1, 2.6%), mediastinum (*n* = 1, 2.6%) and unknown primary (*n* = 1, 2.6%). The most common primary sites in the MT group were the digestive system (*n* = 14, 28.0%), followed by hematopoietic and lymphoid tissues (*n* = 12, 24.0%), female genital organs (*n* = 8, 16.0%), lung (*n* = 8, 16.0%), head and neck (*n* = 4, 8.0%), bladder (*n* = 1, 2.0%) and breast (*n* = 1, 2.0%). No significant difference was found in the primary sites between the CM and MT groups.

The most common histology in the CM group was adenocarcinoma (*n* = 16, 41.0%), followed by malignant tumors of hematopoietic and lymphoid tissues (*n* = 9, 23.1%), squamous cell carcinoma (*n* = 6, 15.4%), small cell carcinoma (*n* = 3, 7.7%), hepatocellular carcinoma (*n* = 2, 5.1%), renal cell carcinoma (*n* = 2, 5.1%), and undifferentiated carcinoma (*n* = 1, 2.6%). The most common histology in the MT group was malignant tumors of hematopoietic and lymphoid tissues (*n* = 14, 28.0%), followed by adenocarcinoma (*n* = 13, 26.0%), squamous cell carcinoma (*n* = 10, 20.0%), hepatocellular carcinoma (*n* = 4, 8.0%), undifferentiated carcinoma (*n* = 3, 6.0%), small cell carcinoma (*n* = 2, 4.0%), adenosquamous carcinoma (*n* = 3, 4.0%), and rhabdomyosarcoma (*n* = 1, 2.0%). No significant difference was found in the prevalence of each histology between the CM and MT groups.

### 3.3. Pathological Findings in the Brain and Blood Vessels of the CM and CI Groups

The pathological findings of both the CM group and CI group are summarized in Table 3. The mean number of brain sections in the CM group (8.6) was significantly lower (*p* = 0.045) than in the MT group (10.9).

**Figure 2:**
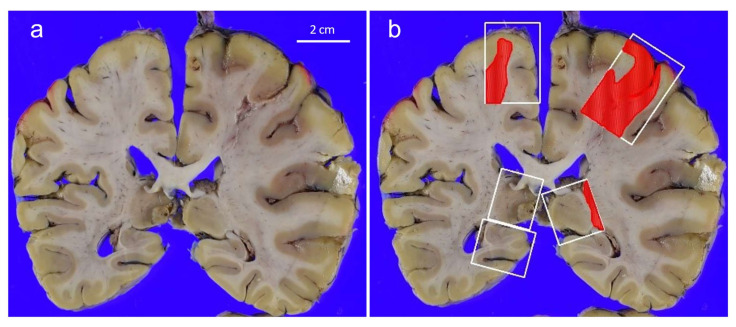
An example of a CM case with multiple CI. (**a**): The cut surface of the brain. The areas of infarctions are unclear. (**b**): Multiple infarctions were identified (red areas) although only restricted sectioned sites could be examined (white rectangles).

The prevalence of multiple CI was almost the same, observed in 76.9% of the CM group and in 79.3% of the CI group cases. Figure 2 shows an example of a CM case with multiple CI. Although not available (NA) sections were sporadically observed, the detailed sites of CI [CM group, CI group] were the following: Cortex/subcortex [71.8%, 66.4%]; basal ganglia/putamen, [83.3% (NA = 3), 82.4% (NA = 2)]; cerebellum [24.2% (NA = 6), 28.3% (NA = 7)]; and brain stem [31.0% (NA = 10), 52.9% (NA = 2)]. Although infarctions in the brain stem tended to be more frequent in the CI group (*p* = 0.066), no significant difference was detected in the sites of CI. 56.4% of the CM group and 50.9% of the CI group exhibited lacunar infarctions only, and no significant difference was found regarding lacunar infarctions (*p* = 0.675).

Hemorrhage was observed in 9 cases (23.1%) in the CM group and 19 cases (35.9%) in the CI group. Massive hemorrhage (≥1.5 cm) was found in 5 cases (12.8%) in the CM group and 12 cases (22.6%) in the CI group. No significant difference was found between groups with regard to hemorrhage.

### 3.4. Mönckeberg’s Atherosclerosis and Amyloid Deposition in Cerebral Vessels

**Figure 3:**
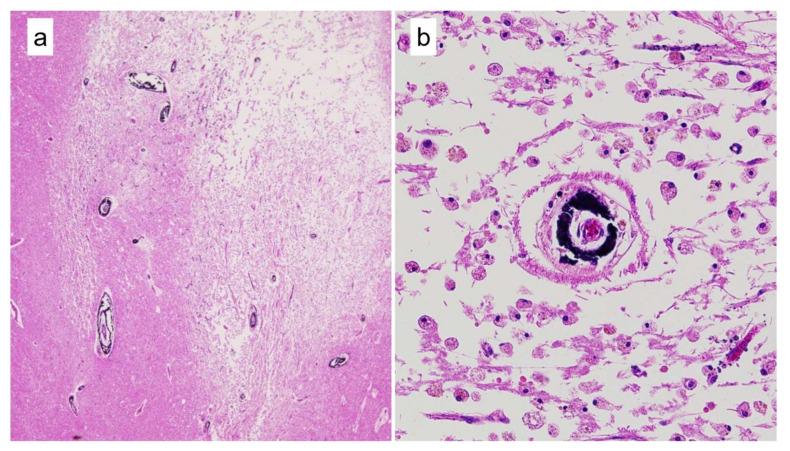
Representative histological images of Mönckeberg’s atherosclerosis in a CM case. (**a**): Much Mönckeberg’s atherosclerosis was observed surrounding the infarction (HE, ×20). (**b**): High magnification image of Mönckeberg’s atherosclerosis (HE, ×200). Calcification that distorted the media spanning the entire circumference was observed.

Through pathological examination, Mönckeberg’s atherosclerosis [12] was often found adjacent to an infarction, especially in CM cases (Figure 3). Thus, we reviewed all available brain sections focusing on Mönckeberg’s atherosclerosis and the results are summarized in Table 4. We analyzed all sizes of arteries that could be microscopically identified in brain sections (maximum: 1799.6 μm in diameter). The diameter of arteries associated with Mönckeberg’s atherosclerosis ranged from 22.6 µm to 410.5 μm. Mönckeberg’s atherosclerosis was found in 15 cases (38.5%) in the CM group, 10 cases (18.9%) in the CI group, and 6 cases (12.0%) in the MT group. 

The frequency of occurrence of Mönckeberg’s atherosclerosis in the CM group was significantly higher than in the MT group (*p* = 0.005), and apparently higher than in the CI group although statistical significance was not attained (*p* = 0.057). No significant difference was observed in the frequency of occurrence of Mönckeberg’s atherosclerosis between the CI and MT groups (*p* = 0.419). Most examples of Mönckeberg’s atherosclerosis were found in the thalamus/basal ganglia and a few cases in the cerebellum, brain stem, and cortex. 

As CKD is generally considered to be associated with Mönckeberg’s atherosclerosis [13,14], we analyzed the frequency of CKD among the three subgroups. The frequency of occurrence of CKD was greatest in the CI group (20.8%) and significantly higher than in the CM group (2.6%, *p* = 0.012) and MT group (2.0%, *p* = 0.004).

Amyloid deposits in vessels were found in two cases (12.0%) in the CM group, four cases (12.0%) in the CI group and two cases (4.0%) in the MT group. No significant difference was found after comparisons of pair combinations among the three groups.

## 4. Discussion

In the present study, we compared the pathology of CI with and without malignancies in three groups of cases, and also focused on intracranial Mönckeberg’s atherosclerosis, which was serendipity acting during our research. The results showed that Mönckeberg’s atherosclerosis occurred in 38.5% cases in the CM group and its incidence was significantly higher than in the MT group (12.0%, *p* = 0.005), and higher than in the CI group (18.9%, *p* = 0.057). These results indicated that intracranial Mönckeberg’s atherosclerosis is a potential risk factor for CI in patients with advanced stage malignancy.

Arteriosclerosis is generally classified into the following two patterns: (i) atherosclerosis having plaque lesions in the intima; and (ii) Mönckeberg’s arteriosclerosis having calcification in the media with or without involvement of the internal elastic [14,15,16]. In the clinical setting, Mönckeberg’s atherosclerosis is frequently identified from inspection of conventional X-ray images of the pelvis or lower extremities [14] and has also been observed in arteries of various organs (coronary arteries [17], temporal arteries [18], lingual artery, facial artery [19], carotid artery [19], uterus [20], breasts [21,22,23], and the upper extremities [24]). 

Although Mönckeberg’s atherosclerosis in various organs has been thoroughly investigated, there has been a paucity of research published on intracranial Mönckeberg’s atherosclerosis. To the best of our knowledge, only a few studies referred to intracranial Mönckeberg’s atherosclerosis, although it should be pointed out that this histology was outside the main purpose of their research objectives [25,26]. Therefore, the clinicopathological characteristics and clinical implications of intracranial Mönckeberg’s atherosclerosis have remained largely unknown. The results of the present study suggest a pressing need for further research into intracranial Mönckeberg’s atherosclerosis.

In our study, Mönckeberg’s’ arteriosclerosis was observed almost exclusively in the basal ganglia. This localization may suggest some hints for the pathogenesis or clinical implications of patients with intracranial Mönckeberg’s atherosclerosis. Regarding calcification in the basal ganglia, the clinical condition, showing bilateral calcification at the basal ganglia, is widely known as Fahr’s disease [27,28]. In addition, many studies on calcification of the basal ganglia are based on imaging findings and its association with dementia [29], Down’s syndrome [30], Alzheimer’s disease [30], and hypoparathyroidism [31]. It has been reported that calcification of the basal ganglia was revealed in head CT scans in 29.7% of 1,133 patients suspected of having had acute ischemic stroke, and was significantly associated with older age and lower BMI scores [32]. These previous reports indicated that basal ganglia tend to be an area where calcium is easily deposited. However, no previous pathologically study examined the association between intracranial Mönckeberg’s atherosclerosis and CI with or without advanced malignant tumor.

Although the pathogenesis of intracranial Mönckeberg’s atherosclerosis is not fully understood, it is considered that vascular smooth muscle cells play an important role in the primarily pathogenesis of medial arterial calcification [16]. It is initiated when vascular smooth muscle cells become damaged and overburdened by hostile conditions in the microenvironment, causing them to lose essential defensive mechanisms and the promotion of transdifferentiation to osteoblast-like cells, which can produce a matrix of bone collagen and noncollagenous proteins. This nidus can then mineralize if the balance of pro-mineralizing factors outweighs the effects of inhibitory factors [16,33].

A strong association between CKD and Mönckeberg’s atherosclerosis has been reported [13,14]. CKD-induced hyperphosphatemia to the rapid development and extensive medial arterial calcification are due in part to mineral dysregulation stemming from the primary renal disorder [14]. Considering this background, we investigated the prevalence of CKD. Contrary to our expectations, only one case (2.6%) had CKD in the CM group, which was comprised of 38.5% cases of Mönckeberg’s atherosclerosis, whereas 20.8% of the CI group had CKD. These results suggest the hypothesis that the pathogenesis of intracranial Mönckeberg’s atherosclerosis is different to that of extracranial Mönckeberg’s atherosclerosis.

Fujita et al. [25] pathologically examined 19 cases with intracranial calcification including 13 cases of neurodegenerative disease. The latter authors classified the calcification into the following three types: type 1, Mönckeberg’s atherosclerosis; type 2, in the parenchyma of the brain; and type 3, along the capillaries. Type 1 calcification was found in all 19 cases in the basal ganglia. Immunohistochemistry of noncollagenous bone matrix proteins (osteopontin, osteocalcin, osteonectin, and bone sialoprotein) revealed that type 1 deposition exhibited a different staining pattern from type 2 and type 3 depositions. The authors’ findings also suggested a unique pathogenesis of intracranial Mönckeberg’s atherosclerosis.

Our findings revealed that the CI group had a significantly higher risk for CI (older age, higher BMI scores, greater prevalence of hypertension and atrial fibrillation, and a higher prevalence of CKD) than those cases in the CM group. These results indicated that the CM group had CI even though the general risk factors were low. On the other hand, no significant difference in general risk factors was observed between the CM and MT groups, whereas the prevalence of intracranial Mönckeberg’s atherosclerosis in the CM group was significantly higher than in the MT group (*p* = 0.005). These findings indicate that intracranial Mönckeberg’s atherosclerosis may be a risk factor for CI in patients with advanced stage malignancy.

The complication of CI severely affects the clinical prognosis of patients with advanced malignancy. To assess adequately the risk of CI in patients with malignancy, it is very important for possible antitumor treatments to be combined with anticoagulant therapy. As the present study suggested that intracranial Mönckeberg’s atherosclerosis is a potential risk factor for CI, we investigated whether intracranial Mönckeberg’s atherosclerosis could be identified in head CT images. However, we could not obtain reliable data because only nine cases were available with head CT scans among cases with Mönckeberg’s arteriosclerosis, although calcification of the basal ganglia was detected by head CT in five cases and four of these five cases could be recognized as vascular calcification.

The limitations of the present study include its retrospective nature, nonuniformity of brain sectioning in pathological work, and the small number of cases. A future prospective study should be conducted to confirm whether blood vessel calcification in the basal ganglia, detected by a head CT, is a risk factor for CI. Our results also should be ideally confirmed by a large-number autopsy series, with systematic sectioning of the basal ganglia and further confirmation of intracranial Mönckeberg’s atherosclerosis with findings in comparative head CT scans.

## 5. Conclusions

The results showed that Mönckeberg’s atherosclerosis was frequently found in the basal ganglia of CM cases, and that intracranial Mönckeberg’s atherosclerosis is a likely risk factor for CI in patients with advanced stage malignancy. Our findings further suggest that the pathogenesis of intracranial Mönckeberg’s atherosclerosis is different from that of extracranial Mönckeberg’s atherosclerosis. As our findings, however, were not unequivocal, further studies are required to confirm our results. The most important interpretation of this study is the potential requirement for further clinical research into intracranial Mönckeberg’s atherosclerosis, which has been a neglected but nevertheless important research topic.

## Figures and Tables

**Table 1:** Clinicopathological characteristics of CI with or without malignancy.

Variable	CM Group *n* = 39	CI Group *n* = 53	*p*-Value
Age, years (mean ± SD)	69.4 ± 9.5	75.0 ± 10.4	0.009
Gender (%)			1.000
Male	22 (56.4%)	31 (58.5%)	
Female	17 (43.6%)	22 (41.5%)	
Body mass index (mean ± SD) ^a^	18.9 ± 0.6	21.0 ± 0.6	0.019
General risk factors for CI			
Hypertension	8 (20.5%)	23 (43.4%)	0.027
Diabetes	5 (12.8%)	9 (17.0%)	0.771
Atrial fibrillation	2 (5.1%)	12 (22.6%)	0.037
Chronic kidney disease	1 (2.6%)	11 (20.8%)	0.012
Infarction of other organs (%) ^b^	16 (41.0%)	28 (52.8%)	0.296
Heart	10 (25.6%)	22 (41.5%)	0.128
Spleen	6 (15.4%)	1 (1.9%)	0.039
Kidney	4 (10.3%)	5 (9.4%)	1.000
Lung	1 (2.6%)	3 (5.7%)	0.635

CI, cerebral infarction; CM, cerebral infarction with malignancy. ^a^ Body mass index was not available in three cases in the CM group. ^b^ The infarct site overlaps in some cases; only the main organs of infarction are listed.

**Table 2:** Clinicopathological characteristics of malignant cases with or without CI.

Variable	CM Group *n* = 39	MT Group *n* = 50	*p*-Value
Age, years (mean ± SD)	69.4 ± 9.5	64.0 ± 14.7	0.052
Gender (%)			
Male	22 (56.4%)	28 (56.0%)	1.000
Female	17 (43.6%)	22 (44.0%)	
Body mass index (mean ± SD) ^a^	18.9 ± 3.8	19.6 ± 3.2	0.398
General risk factors for CI			
Hypertension	8 (20.5%)	7 (14.0%)	0.569
Diabetes	5 (12.8%)	5 (10.0%)	0.743
Atrial fibrillation	2 (5.1%)	2 (4.0%)	1.000
Chronic kidney disease	1 (2.6%)	1 (2.0%)	1.000

CI, cerebral infarction; CM, cerebral infarction with malignancy; MT, malignant tumor. ^a^ Body mass index was not available for three cases in the MT group.

**Table 3:** Pathological findings of brain and vessels in cerebral infarction cases with or without malignancy.

Variable	CM Group *n* = 39	CI Group *n* = 53	*p*-Value
Number of brain sections (mean, range)	8.6 (2–22)	10.9 (4–25)	0.045 *
Number of brain infarctions			0.803
Multiple	30 (76.9%)	42 (79.3%)	
Single	9 (23.1%)	11 (20.8%)	
Site of cerebral infarction (%)			
Cortex/Subcortex	28 (71.8%)	35 (66.4%)	0.652
Thalamus/Basal ganglia	30/36 (83.3%), NA = 3	42/51 (82.4%), NA = 2	1.000
Cerebellum	8/33 (24.2%), NA = 6	13/46 (28.3%), NA = 7	0.799
Brain stem	9/29 (31.0%), NA = 10	27/51 (52.9%), NA = 2	0.066
Lacunar infarction (<1.5 cm) only (%)			0.675
Yes	22 (56.4%)	27 (50.9%)	
No	17 (43.6%)	26 (49.1%)	
With hemorrhage	9 (23.1%)	19 (35.9%)	0.253
Micro hemorrhage (<1.5 cm)	4 (10.3%)	7 (13.2%)	0.754
Massive hemorrhage	5 (12.8%)	12 (22.6%)	0.284

CM, cerebral infarction with malignancy; CI, cerebral infarction; NA, not available; * *p* < 0.05

**Table 4:** Status of Mönckeberg’s atherosclerosis and amyloid deposition in cerebral blood vessels.

Variable	CM *n* = 39	CI *n* = 53	MT *n* = 50	*p*-Value (CM vs. CI)	*p*-Value (CM vs. MT)	*p*-Value (CI vs. MT)
Mönckeberg’s atherosclerosis	15 (38.5%)	10 (18.9%)	6 (12.0%)	0.057	0.005	0.419
Cortex/Subcortex	1	0	0	0.424	0.438	-
Thalamus/Basal ganglia	14	10	6	0.056	0.009	0.416
Cerebellum	2	0	1	0.171	0.586	0.465
Brain stem	1	0	0	0.363	0.387	-
Amyloid deposition at vessels	2 (5.1%)	4 (7.6%)	2 (4.0%)	1.000	1.000	0.679
Chronic kidney disease	1 (2.6%)	11 (20.8%)	1 (2.0%)	0.012	1.000	0.004

CM, cerebral infarction with malignancy group; CI, cerebral infarction group; MT, malignant tumor group. Multiple Mönckeberg’s atherosclerosis was separately categorized.

## Data Availability

The data presented in this study are available on request from the corresponding author.
